# A Criticism

**Published:** 1889-05

**Authors:** 


### A Criticism.

BY C. M.

In the January number of the DENTAL REGISTER there is reprinted an article from the Lockport *American* a criticism of an article upon Aluminium in the Springfield *Union*, signed " C. M." As the sentiments in that article of the *Union* tally as closely with my own as can be expressed in print, I have to take up the defence of " C. M." The editor spoke about Dr. Waldo's " experience and scientific knowledge." Well, let us analize his criticism a little: His authority for spelling " Aluminum" is certainly not acknowledged in scientific circles because the right of priority to name a thing is in the discoverer, and Wohler, who first discovered the metal had the right to name it, and he called it " Aluminium;" that settles this point sufficiently for any scientific investigation.

If Dr. Waldo never saw aluminium tarnish he must be very blind. I have aluminium in sheets, wire, and ingots and they are all as heavily tarnished as zinc; only we must not look upon it with prejudiced eyes, which do not see any thing. " C. M." did not say, that it is attacked by sulphurretted hydrogen, and every chemist knows that. Therefore Dr. Waldo made no point by emphasizing that statement. How can a man of "experience and scientific knowledge" make a statement like: "There is no reason for thinking that common salt, at least table salt, would have the slightest action on aluminum." Facts in chemistry are not settled by "reasons to think," but by the experiment. Let him boil his aluminum sheet in a solution of salt, such as would be done when used for cooking and "his reason for thinking" would become a fact one way or other; he will find that salt does take away from aluminium and oxidize it enough to make it not a whit superior to zinc.

"It is not attacked by nitric acid nor by sulphuric acid unless they are very strong" is true in the very reverse; both acids diluted, attack it almost as rapidly as zinc, but have very little action upon it when very strong, just like iron and steel, neither of which is acted upon by these acids when strong. He admits that hydrochloric acid will attack it, and vinegar and salt will also attack it. Why, I thought there was "no reason to think" that salt would act on it!

I would suggest to "Dr. Waldo's experience and scientific knowledge" a way in which we do things in chemistry.

We take a sheet of aluminium, weigh it carefully and submit it to the tests; weigh it again afterward and find the effect; an increase or decrease shows attack. We have found field glasses, the tubes of which were made of common brass which had been used for years, and beyond a slight oxidization or sulphurization, had not deteriorated; just as aluminium tubes in opera-glasses which only show a slight coating of white aluminium oxide.

How about soapsuds? Common, plain soapsuds, and washing compounds eat up aluminium faster than any other metal that is used. I have some excellent 10 per cent. aluminium bronze which has been in a drawer together with some brass, common bronze, phosphor bronze, etc., and it is the worse looking of the whole lot. It was made of pure copper and pure aluminium; is very hard; the thought of drawing it into wire or sheets has to be abandoned, because as any one can find by a trial, it would'nt do it. In connection with this I would mention a certain fallacy, we may call it euphemistically, inaccuracy in speaking of alumi nium alloys, "aluminium bronze" which is drawn out into wire and shows some good properties, is, in reality an aluminium brass with a very small percentage of aluminium and it seems to improve, indeed the quality of the brass, but the percentage is not as material as the name would suggest.

Without entering into much of Dr. Waldo's assertion about alloys, which is correct, why does it happen that the price of aluminium keeps at $10 wholesale, when there are so many people who make it at so much less? The article by "C. M." does not speak of alloys, but only the pure aluminium, therefore, any excuse of defects of aluminium by mentioning its good properties in alloys does not affect these defects. When the article appeared it spoke about the aluminium works that existed, those that were being erected were as numerous as there are cities and aluminium companies in the civilized world. If we could get rid, conveniently, of a considerable number of pounds of aluminium we would like to buy it at 20 shillings a pound but we are afraid that the capacity of the world wouldn't amount to 10,000 pounds even at that price.

